# Development of a preclinical model of donation after circulatory determination of death for translational application

**DOI:** 10.1186/2047-1440-3-13

**Published:** 2014-06-14

**Authors:** Géraldine Allain, Thomas Kerforne, Rodolphe Thuret, Pierre-Olivier Delpech, Thibaut Saint-Yves, Michel Pinsard, Thierry Hauet, Sébastien Giraud, Christophe Jayle, Benoît Barrou

**Affiliations:** 1 INSERM U1082, CHU de Poitiers, rue de la Milétrie, B.P. 577, F-86021 Cedex Poitiers, France; 2 CHU de Poitiers, Service de Chirurgie cardio-thoracique, Poitiers F-86000, France; 3 CHU de Poitiers, Service de Réanimation chirurgicale, Poitiers F-86000, France; 4 CHU de Montpellier, Service d’Urologie et de transplantation rénale, Montpellier F-34295, France; 5 CHU de Poitiers, Service d’Urologie, Poitiers F-86000, France; 6 Université de Poitiers, Faculté de Médecine et de Pharmacie, Poitiers F-86000, France; 7 CHU Poitiers, Service de Biochimie, Poitiers F-86000, France; 8 IBISA Platform ‘Experimental Surgery and Transplantation’, INRA, Domaine expérimental du Magneraud, Surgères F-17700, France; 9 GH Pitié-Salpêtrière, AP-HP, Service d’Urologie et de transplantation rénale, Paris F-75013, France; 10 UPMC Université Paris VI, Paris F-75013, France

**Keywords:** Animal model, Donation after circulatory determination of death, Extracorporeal membrane oxygenation, Ischemia/reperfusion injury, Organ donor management

## Abstract

**Background:**

Extracorporeal membranous oxygenation is proposed for abdominal organ procurement from donation after circulatory determination of death (DCD). In France, the national Agency of Biomedicine supervises the procurement of kidneys from DCD, specifying the durations of tolerated warm and cold ischemia. However, no study has determined the optimal conditions of this technique. The aim of this work was to develop a preclinical model of DCD using abdominal normothermic oxygenated recirculation (ANOR). In short, our objectives are to characterize the mechanisms involved during ANOR and its impact on abdominal organs.

**Methods:**

We used Large White pigs weighing between 45 and 55 kg. After 30 minutes of potassium-induced cardiac arrest, the descending thoracic aorta was clamped and ANOR set up between the inferior vena cava and the abdominal aorta for 4 hours. Hemodynamic, respiratory and biochemical parameters were collected. Blood gasometry and biochemistry analysis were performed during the ANOR procedure.

**Results:**

Six ANOR procedures were performed. The surgical procedure is described and intraoperative parameters and biological data are presented. Pump flow rates were between 2.5 and 3 l/min. Hemodynamic, respiratory, and biochemical objectives were achieved under reproducible conditions. Interestingly, animals remained hemodynamically stable following the targeted protocol. Arterial pH was controlled, and natremia and renal function remained stable 4 hours after the procedure was started. Decreased hemoglobin and serum proteins levels, concomitant with increased lactate dehydrogenase activity, were observed as a consequence of the surgery. The serum potassium level was increased, owing to the extracorporeal circulation circuit.

**Conclusions:**

Our ANOR model is the closest to clinical conditions reported in the literature and will allow the study of the systemic and abdominal organ impact of this technique. The translational relevance of the pig will permit the determination of new biomarkers and protocols to improve DCD donor management.

## Background

Transplantation is the best alternative to end-stage organ disease [1]. The increasing number of patients waiting for an organ has led transplant teams to consider new sources of organs. A possible answer to this organ shortage is the use of organs from donation after circulatory determination of death (DCD) [2]. Cardiac arrest may be unexpected (type I and II of the Maastricht classification), or occur after life-sustaining care withdrawal (type III of the Maastricht classification). One of the challenges of DCD management is to correct or prevent ischemia-reperfusion injury due to the unavoidable period of warm ischemia. Warm ischemia is usually more pronounced in uncontrolled donation and it is of utmost importance to define an effective method to limit it. As this method will be applied after death certification in uncontrolled donors, it will not raise the usual ethical concerns that can be encountered in controlled donation.

In renal transplantation centers using kidneys from DCD, it has been shown that this organ source could increase the donor pool by 20 to 40% [3]. However, organs from these donors are more prone to ischemia-reperfusion injury because DCD kidneys suffer a period of warm ischemic damage (no-flow and low-flow periods) in addition to cold ischemia during *ex-vivo* conservation. Consequently, the risk of primary nonfunction and delayed graft function is higher [4,5].

Methods for conditioning abdominal organs before removal have evolved. Initially, protocols were limited to *in-situ* cooling using an intra-aortic double balloon catheter or Gillot’s catheter [6,7]. This technique is referred to as cold *in-situ* perfusion. Since the middle of the 1990s, normothermic extracorporeal membrane oxygenation has been proposed, either in hypothermic conditions for total body cooling or in normothermic conditions limited to the abdomen using abdominal normothermic oxygenated recirculation (ANOR). This method has been developed and applied by several groups around the world [8-12]. This technique uses a normothermic extracorporeal membrane oxygenation circuit with cannulae introduced by a femoral approach and an occlusive balloon positioned in the descending thoracic aorta to limit circulation to the abdominal region.

**Figure 1:**
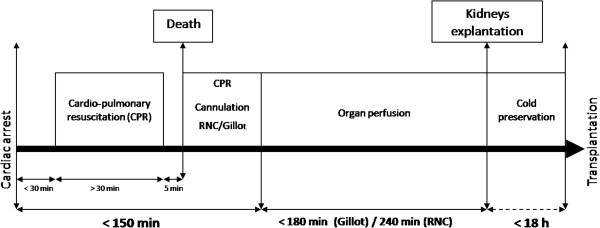
**Recommendations for kidney removal from donation after cardiac death according to the French national regulatory authority.** ANOR, abdominal normothermic oxygenated recirculation.

In France, a national protocol was designed to use exclusively uncontrolled donors (DCD type II of the Maastricht classification). The program started in 2007 and the protocol is summarized in Figure 1[13]. In 2000, a team from Barcelona compared kidney preservation with cold *in-situ* perfusion, hypothermic cardiopulmonary bypass, and ANOR [8]. A significant decrease in primary nonfunction and delayed graft function with satisfactory long-term graft survival was identified for the ANOR group. Several other groups have reported their experiences with ANOR in uncontrolled and controlled donors and suggested the superiority of ANOR in DCD, as compared with cold *in-situ* perfusion (for a review, see [14]). However, no formal demonstration has been established.

Many issues remain to be addressed regarding the ideal protocol, such as ideal temperature and optimal duration. The use of additives is also a pivotal question. Clearly, experimental work is mandatory to better define the optimal conditions of this technique, which will help to limit or even repair the injuries induced by warm ischemia. The aim of our work was to develop a preclinical model of DCD based on the current recommendations of the French national regulatory authority on the study of kidneys exposed to ANOR and to optimize the conditions under which the technique is used.

## Methods

### Experimental model

The protocol was carried out in accordance with the regulations of the French Ministry of Agriculture, the National Institute for Agronomic Research and the regional ethics committee on animal experiments (Comité d’éthique pour l’expérimentation animale en Poitou-Charentes). The animals were Large White male pigs about 3 months old and weighing between 45 and 55 kg. These animals have been used in the experimental surgery laboratory for 15 years to study ischemia-reperfusion injuries with renal transplantation models [15,16]. The pig is well suited for preclinical studies because its anatomy and physiology are very similar to those of human beings and it has rapidly been chosen for potential applications in clinical practice [17].

We used a Rotaflow Maquet centrifugal pump with Softline Coating Maquet-Jostra pre-heparinized circuit (Maquet Cardiopulmonary AG, Hirrlingen, Germany). A thermoregulatory system is connected to maintain a temperature of 37°C. The circuit is purged with hydroxyethylamide (Restorvol® 6%, Fresenius Kabi, Sèvres, France). The priming is approximately 700 to 800 cm^3^.

### Anesthetic procedure

Induction of the animal is performed with a Hunter mask with a mixture of nitrous oxide and oxygen 50/50 associated with 8% sevoflurane then 2.5% isoflurane. A perfusion line is set at a marginal vein of the ear. Orotracheal intubation is performed under laryngoscopic control. Controlled ventilation is performed with a respiratory rate of 16/min while maintaining expired CO_2_ between 35 and 45 mmHg. Curarization of the animal is performed by initial injection of 8 mg pancuronium bromide followed by reinjection adapted to the behavior of the animal. Intraoperative analgesia is achieved by intravenous injections of 20 mg nalbuphine and 20 mg nefopam.

### Surgical procedure

**Figure 2:**
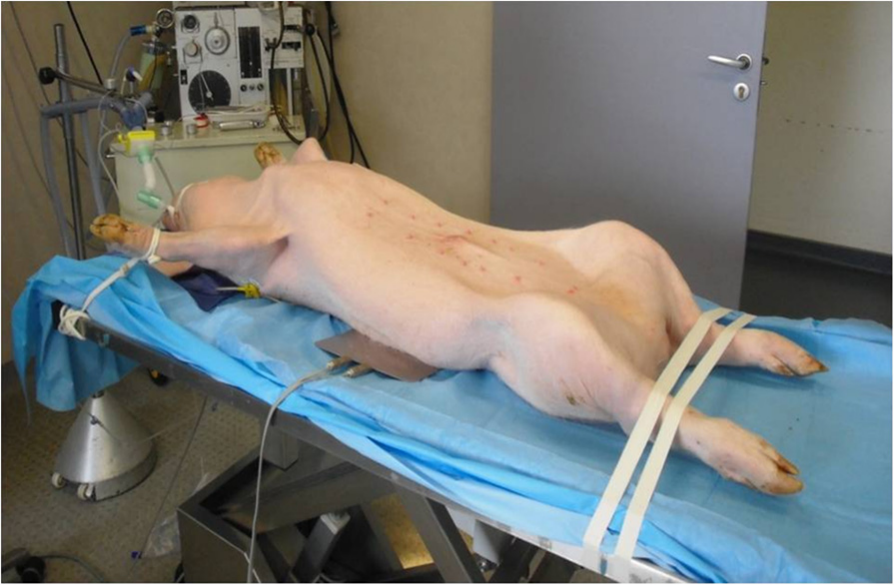
Installation of the animal for the procedure.

**Figure 3:**
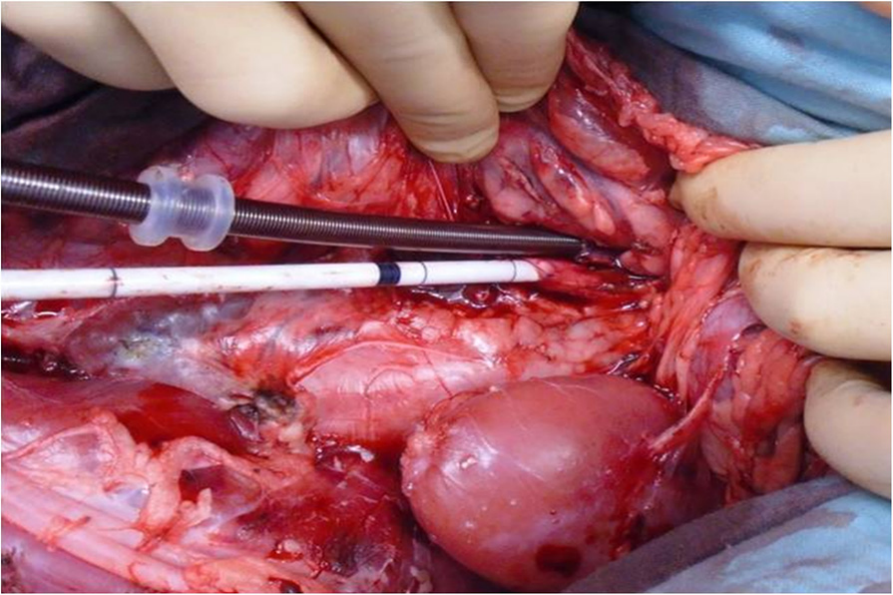
Cannulation of aorta and inferior vena cava under renal vessels by an extraperitoneal approach.

**Figure 4:**
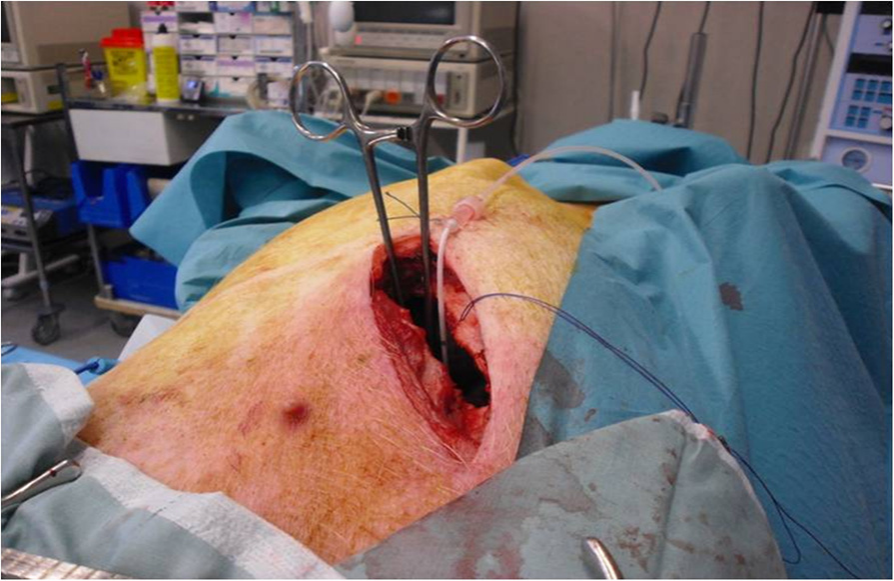
Aortic clamping and arterial catheter through left thoracotomy.

**Figure 5:**
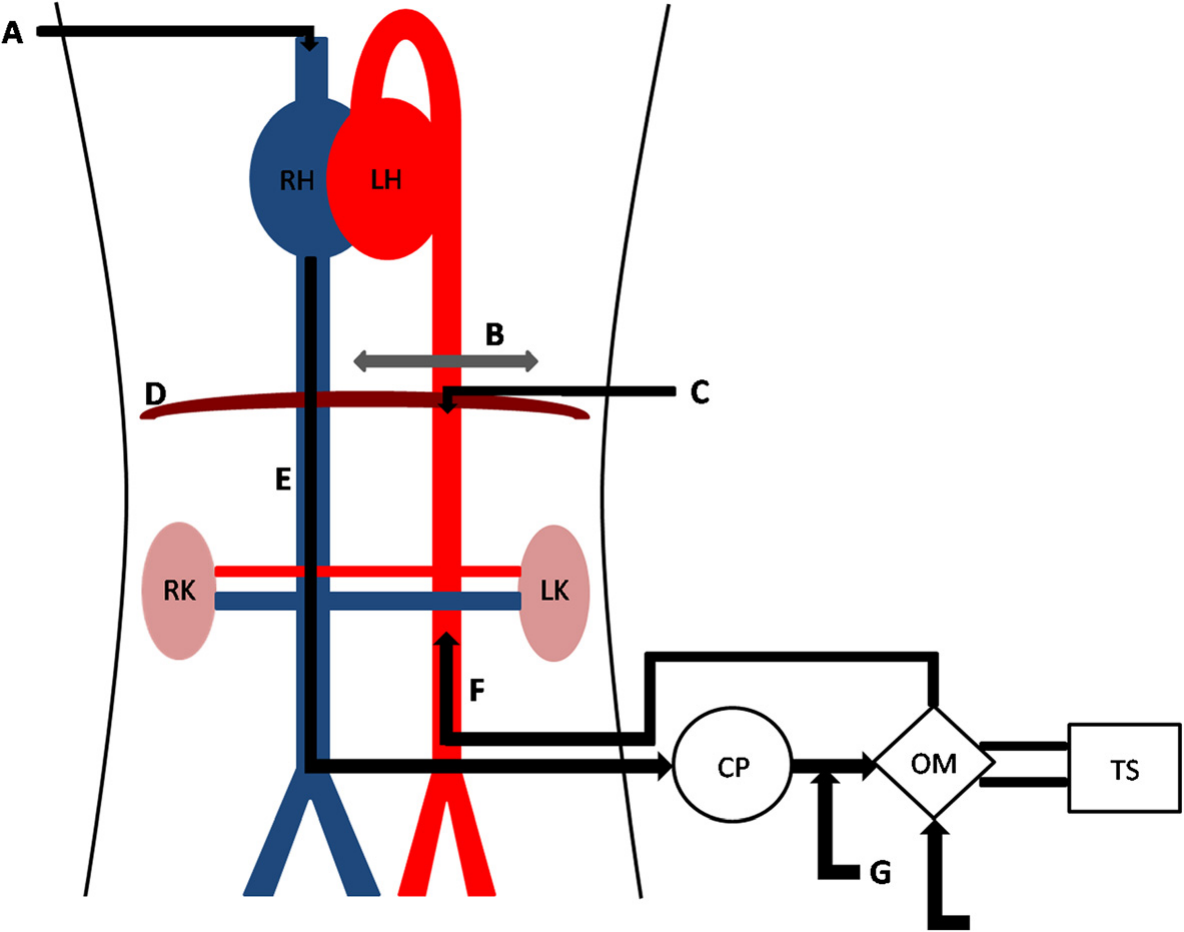
**Abdominal normothermic oxygenated recirculation in the preclinical porcine model.** A, central venous catheter; B, aortic clamp; C, arterial catheter; CP, centrifugal pump; D, diaphragm; E, venous canulae in inferior vena cava; F, arterial canulae in aorta; G, perfusion entry; H, gas entry; LH, left heart; LK, left kidney; OM, oxygenation membrane; RH, right heart; RK, right kidney; TS, thermoregulatory system.

A video of the procedure is provided in Additional file 1. The animal is placed supine with access to the cervical, thoracic, and abdominal regions (Figure 2). First, a central venous catheter is positioned in the internal jugular vein after right cervicotomy. Then, laparotomy is performed. The abdominal aorta and inferior vena cava under the renal vessels are dissected, since the femoral vessels are too small to receive cannulae of sufficient size to ensure the flow rate used. We chose an extraperitoneal approach to avoid lymphatic losses and to limit vascular filling and hemodilution. Indeed, the formation of a third sector is frequently encountered in renal transplants performed by a transperitoneal approach in the laboratory. A bolus of 300 IU/kg of unfractionated heparin is injected through the central venous catheter. This dosage is recommended by the French national regulatory authority. After 5 minutes, a 14 Fr arterial cannula (Medtronic Inc., Minneapolis, MN) is placed in the abdominal aorta under the renal arteries and a 21 Fr long multiperforated venous cannula (Medtronic Inc., Minneapolis, MN) is positioned at the junction of the inferior vena cava and the right auricle (Figure 3). Cannulae are introduced according to the Seldinger technique, so as to be completely sealed.Injection of 2 g potassium chloride by the central venous catheter leads to cardiac arrest. Mechanical ventilation is stopped. The descending thoracic aorta is approached by a short left thoracotomy so as to be clamped (Figure 4). Another catheter is placed downstream from the clamp to monitor blood pressure. The laboratory model is shown schematically in Figure 5. After 30 minutes of warm ischemia, ANOR is started for 4 hours.

Abdominal normothermic oxygenated recirculation:

### Procedure, progression, and data collection

Monitoring procedures with objectives and corrective actions are summarized in Table 1. Blood samples are collected via the central venous catheter before laparotomy and every hour during the ANOR on the arterial line. Plasmas from heparinized blood are stored for later biochemical analysis. At the end of 4 hours, kidneys are explanted and stored for later analysis. The ANOR is stopped. Cannulae and catheters are removed and the animal is euthanized.

**Table 1:** Monitoring procedures during abdominal normothermic oxygenated recirculation

**Monitored parameters**	**Objectives**	**Type of monitoring**	**Corrective actions**
Pump flow rate	2.5 to 3 l/min	Continuous monitoring	Vascular filling: saline and hydroxyethyl starch (10 ml/(kg h))
Mean arterial pressure	>60 mmHg		Continuous infusion of noradrenalin on the circuit
Central venous pressure	<5 mmHg		—
pH	7.5	Blood samples on the circuit every 30 minutes	Continuous infusion of sodium bicarbonate on the circuit
Partial pressure of carbon dioxide	30 to 40 mmHg		Sweeping gas flow adjustment
Partial pressure of oxygen	100 to 150 mmHg		Fraction of inspired oxygen adjustment
Activated clotting time	>200 s		Bolus of 5000 IU unfractionated heparin on the circuit

### Parameters of monitoring procedures

The pump flow rate was monitored continuously during the procedure. The arterial pressure and central venous pressure were measured continuously during ANOR using a monitor HP 56S (Hewlett-Packard, Les Ulis, France). The partial pressures of oxygen and carbon dioxide, the fraction of inspired oxygen, and the pH were measured every 30 minutes during the procedure, using an i-STAT portable clinical analyzer (Hewlett-Packard, Les Ulis, France). The activated clotting time was also measured every 30 minutes, using a Hemostasis Management System (Medtronic, Boulogne-Billancourt, France).

Levels of plasma potassium, sodium, protein, lactate dehydrogenase (as an acidosis marker), and blood hemoglobin were quantified every hour during the ANOR procedure using an automated chemistry analyzer (Modular, Roche Diagnostics, France). To determine renal function, plasma creatinine was measured every hour during the ANOR, again using an automated chemistry analyzer (Modular, Roche Diagnostics, France).

### Statistical analysis

Results are expressed as mean ± standard deviation. For the statistical analysis among groups, we used NCSS software (NCSS LLC, USA) and ANOVA or two-sample *t* test analysis in the case of normality (skewness, kurtosis, and omnibus tests) and equality of variance (modified Levene equal-variance test) and the Mann–Whitney *U* test or Kruskal-Wallis test for multiple comparison analysis where these parameters were not met. Differences with a *P* value of less than 0.05 were considered significant.

## Results and discussion

### Monitored parameters during abdominal normothermic oxygenated recirculation

Six 4-hour ANOR procedures were performed. Animal weight varied between 45 and 55 kg. The monitored parameters and objectives are listed in Table 1.

Results for the monitored parameters are expressed as mean ± standard deviation (Table 2). The pump flow rate, mean arterial pressure, and central venous pressure were controlled continuously during the ANOR procedure. Partial pressures of oxygen and of carbon dioxide were stabilized during the procedure, between 122 and 173 mmHg and between 30 and 37 mmHg, respectively. There was an exception for activated clotting time, which was rarely above 200 s. It was therefore often necessary to inject 5000 IU unfractionated heparin every 30 minutes during the ANOR process. The objectives, listed in Table 1, were achieved under reproducible conditions.

**Table 2:** Comparison of monitored parameters during abdominal normothermic oxygenated recirculation (mean ± standard deviation)

	**ANOR 1**	**ANOR 2**	**ANOR 3**	**ANOR 4**	**ANOR 5**	**ANOR 6**
Weight (kg)	49	51	55	45	50	45
Pump flow rate (l/min)	2.5 ± 0.03	2.5 ± 0.03	2.8 ± 0.03	2.7 ± 0.05	2.6 ± 0.05	2.5 ± 0.03
Mean arterial pressure (mmHg)	65 ± 6	64 ± 5	84 ± 3	86 ± 10	77 ± 3	75 ± 6
Noradrenalin (μg/(kg min))	0.7 ± 0.05	1.1 ± 0.19	0.6 ± 0.08	0.6 ± 0.2	0.6 ± 0.07	0.7 ± 0.15
Fraction of inspired oxygen (%)	56 ± 3	62 ± 5	59 ± 5	45 ± 2	54 ± 1	56 ± 3
Partial pressure of oxygen (mmHg)	123 ± 25	122 ± 16	141 ± 27	173 ± 31	149 ± 13	151 ± 19
Sweeping gas flow (l/min)	3.2 ± 0.2	4 ± 0	4.3 ± 0.2	3.3 ± 0.2	3.6 ± 0.1	3.5 ± 0
Partial pressure of carbon dioxide (mmHg)	37 ± 5	35 ± 1	35 ± 1	32 ± 2	30 ± 1	31 ± 1
Activated clotting time (s)	174 ± 32	183 ± 27	180 ± 21	198 ± 33	173 ± 20	171 ± 20

ANOR, abdominal normothermic oxygenated recirculation.

### Evolution of biological parameters during abdominal normothermic oxygenated recirculation

The evolution of biological parameters during ANOR is summarized in Table 3. There was no significant difference between T0 and 4 hours of ANOR for pH (7.4 ± 0.01 to 7.4 ± 0.03, respectively), serum sodium concentration (138 ± 1 to 138 ± 1 mmol/l, respectively), serum creatinine concentration (99 ± 6 to 129 ± 27 μmol/l, respectively). Lactate dehydrogenase activity was significantly increased between T0 and 4 hours (714 ± 48 to 1041 ± 204 IU/l, respectively), and was probably related to the stresses of the surgery and muscular attrition. Serum protein and hemoglobin levels showed significant decrease between T0 and 4 hours of ANOR, demonstrating hemodilution and bleeding during surgery (56 ± 3 to 37 ± 3 g/l for serum protein concentration and 12 ± 0.1 to 9.3 ± 0.6 g/dl for hemoglobin level). Correction of serum potassium levels was not perfect, with lower levels at 4 hours than at 1 hour; however, they remained significantly higher than at T0. This was due to the extracorporeal circulation circuit.

**Table 3:** Evolution of biological parameters during abdominal normothermic oxygenated recirculation (means ± standard deviation)

	**T0**	**1 hour**	**2 hours**	**3 hours**	**4 hours**	***P *** **T0 versus 4 hours**
pH	7.4 ± 0.01	7.42 ± 0.06	7.42 ± 0.03	7.40 ± 0.05	7.44 ± 0.03	0.1795
Serum potassium (mmol/l)	4.1 ± 0.2	7.1 ± 0.5	7.9 ± 0.3	7.3 ± 0.5	6.8 ± 0.6	0.0002*
Serum sodium (mmol/l)	138 ± 1	136 ± 1	139 ± 1	137 ± 2	139 ± 3	0.4825
Serum protein (g/l)	56 ± 3	41 ± 3	45 ± 2	39 ± 2	37 ± 3	0.0002*
Hemoglobin (g/dl)	12 ± 0.1	10.9 ± 0.5	11.1 ± 0.5	10.1 ± 0.8	9.3 ± 0.6	0.0001*
Creatinine (μmol/l)	99 ± 6	130 ± 5	132 ± 18	133 ± 21	129 ± 27	0.1673
Lactate dehydrogenase (IU/l)	714 ± 48	1,036 ± 193	1,354 ± 133	1,132 ± 233	1,041 ± 204	0.0424*

*: statistically significant.

## Discussion

The increasing demand for transplantation has created a need for additional organ sources, and acceptance criteria have been widened, not only for kidney retrieval but increasingly for other organs with a lower tolerance of warm ischemia, such as the liver, pancreas, and lungs [18,19]. This expansion of donor criteria to include the use of grafts from elderly donors, DCD, or organs from donors with other medical conditions has underlined the need for a technique that prevents any further damage during the preservation period. Previous reports strongly suggest that DCD donors are a valuable source of organs [3,14,20,21]. Reconditioning using ANOR appears to give better results than *in-situ* cooling preservation, with a reduction of primary nonfunction and delayed graft function [8]. In France, the national regulatory authority (Agence de la Biomédecine) has defined the conditions for organ removal, specifying the tolerated durations of warm and cold ischemia, for uncontrolled donors (type II of the Maastricht classification). However, no preliminary study has determined the optimal conditions for ANOR. Consequently, there is room for experimental studies to decipher the different steps of the protocol.

The development of a porcine preclinical experimental model of DCD donors allows for a study of the ANOR impact on organs without the limitations of a clinical study. Several models of DCD donors have previously been published in the literature to study the conditioning of kidney transplants by normothermic perfusion. Recently, using rats of 300 g and a warm ischemia period corresponding to clamping of the renal artery for 15 or 30 minutes, Moers *et al.* studied reperfusion for 1 to 2 hours [22]. However, the restored pulsatile flow was far from comparable to a continuous flow of ANOR. In 2008, Hosgood *et al.* used a Large White pig model weighing between 40 and 50 kg [23]. In this model, after 25 minutes of warm ischemia, kidneys were reperfused normothermically for 3 hours in an isolated organ preservation system using autologous blood. However, this experimental condition remains far from a clinical situation. In 2010, Rojas-Pena *et al.* used an extracorporeal membrane oxygenation circuit with pigs from 25 to 30 kg [24]. Both external jugular veins were cannulated in place of the inferior vena cava. In addition, the absence of clamping of the descending thoracic aorta mimicked a systemic circulation model, which was not exactly the ANOR condition. The durations of warm ischemia were 10 and 30 minutes, respectively, and 90 and 100 minutes for reperfusion.

To our knowledge, the uncontrolled DCD model presented herein is the closest to the conditions encountered in clinical practice. Indeed, we use a large animal, the pig, well accepted as a preclinical model with a size, anatomy, and cardiovascular physiology very similar to those of human beings. Our model also used truly regional circulation between the inferior vena cava and the abdominal aorta, which was limited to the subdiaphragmatic region by clamping of the descending thoracic aorta. Moreover, our model is highly reproducible. In all cases, hemodynamic and respiratory objectives were achieved with very similar conditions from one animal to another. After 4 hours of ANOR, there was no significant difference compared with T0 for pH and plasma sodium levels. Lactose dehydrogenase levels were increased, a sign of tissue damage that was probably due to the stresses of surgery (incisions, muscular attrition, and so on); the lack of change between 1 and 4 hours of ANOR highlight the fact that this marker is not adapted for discriminant diagnosis determination. We observed an expected hemodilution, mainly due to the ANOR priming (about 700 to 800 cm^3^). However, hemoglobin levels remained correct, at 9.3 ± 0.6 g/dl after 4 hours of ANOR. Interestingly, in our hands, renal function likewise remained stable during ANOR and reached a steady state during the procedure.

However, the injection of heparin before cardiac arrest is a critical limit in this model. We aim to correct this easily in future protocols. Note that in previously described models, injection of heparin was likewise carried out before cardiac arrest.

It would also have been interesting to monitor urine production during ANOR. However, bladder catheterization of Large White male pigs is not possible, owing to a tortuous urethra and we consequently chose not to approach the bladder surgically, thereby avoiding the opening of the peritoneum and fluid, particularly lymph fluid, accumulation. However, we noted that at the end of ANOR, urine (about 500 cm^3^) was collected in the bladder. To better monitor kidney function, the implantation of a urine catheter during ANOR is envisioned for future experiments.

## Conclusions

We developed a preclinical experimental model of uncontrolled DCD kidneys donors using ANOR. This model will allow the development of experimental procedures and the study of systemic and renal impact of the ANOR at different steps. Many issues could be addressed, such as optimal temperature and duration. This model could help to tailor strategies and to build clinical protocols for preservation and viability prediction and evaluate preservation protocols following ANOR. It is ideal to determine the optimal conditions for pre- and post-conditioning, which hold the potential to reduce graft injury. It will allow comparative studies between ANOR and *in-situ* cooling preservation. This model can also facilitate the study of conditioning protocols for other abdominal organs with ANOR and the effect of additives on ischemia-reperfusion injury. The translational relevance of the pig also permits the study of relevant biomarkers for real-time evaluation of organ viability during and after ANOR. Finally, the technology is readily available and easy to handle with a relatively short learning curve. Such programs could be included in transplantation operative procedures and surgical skills taught to young European surgeons entering the field of transplantation surgery.

## Abbreviations

ANOR: abdominal normothermic oxygenated recirculation; ANOVA: analysis of variance; DCD: donation after circulatory determination of death.

## Competing interests

The authors declare that they have no competing interests.

## Authors’ contributions

GA wrote the article, participated in the development of the model, realized the surgical protocol, and participated in the monitoring procedure. TK participated in the development of the model, conducted anesthesia, and participated in the monitoring procedure. RT participated in the development of the model. POD and TSY participated in the surgical protocol and in the monitoring procedure. MP participated in the development of the model and the anesthetic procedure. TH supervised the work and contributed to the writing. SG contributed to the writing. CJ participated in the development of the model, the realization of the surgical protocol, and the monitoring procedure. BB contributed to the development of the model and to the writing. All authors read and approved the final manuscript.

## Supplementary Material

Additional file 1 Video of surgical procedure. Click here for file
